# Spontaneous fragmentation of JJ stent can occur as early as 3 weeks post‐insertion: Case report

**DOI:** 10.1002/ccr3.3444

**Published:** 2020-11-20

**Authors:** Fayez T. Hammad

**Affiliations:** ^1^ College of Medicine and Health Sciences United Arab Emirates University and Mediclinic Al Ain Al Ain UAE

**Keywords:** case report, early, JJ stent, spontaneous fragmentation

## Abstract

Spontaneous JJ stent fragmentation can occur as early as 3 weeks post‐insertion. This could be due to a hostile urine environment and might be avoided by strict dietary modification, adequate hydration, and close follow‐up.

## Graphical abstract

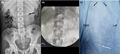

## 1 BACKGROUND

Spontaneous JJ stent fragmentation (SSF) is rare and usually occurs in forgotten or neglected stents. We present two cases of SSF which occurred 3 and 5 weeks post‐insertion. This could be due to a hostile urine environment and might be avoided by strict dietary modifications, adequate hydration, and close follow‐up.

The use of double J (JJ) ureteric stent in its current form is associated with several short‐ and long‐term complications.^1^ Spontaneous stent fragmentation (SSF) is a very rare but previously described complications.^2^, ^3^, ^4^, ^5^, ^6^, ^7^, ^8^, ^9^, ^10^ This potentially serious complication increases patients’ morbidity and requires further procedures to remove the retained fragments. The exact reason for SSF is unclear but has been attributed to the stent material, hostile and harsh urine environment, and the duration of leaving the stent inside the body.^10^, ^11^, ^12^ The vast majority of the previously reported cases were associated with calcifications and encrustation of stents which had been neglected or forgotten for longtime.^4^, ^5^, ^6^, ^9^ Herein, we report, for the first time, on two cases in which the SSF has occurred as early as 3 and 5 weeks post‐insertion and reflect on the possible causes and ways to prevent this early complication.

## 2 CASE PRESENTATION

### 2.1 Case‐1

A 39‐year‐old construction laborer presented to our facility with sudden and severe exacerbation of JJ stent‐related symptoms of dysuria and urinary frequency. Three weeks earlier, he had presented to another healthcare facility for the first time with bilateral flank pain and dysuria associated with nausea of 3 days duration. There was no history of fever or previous urological diseases or interventions. He had an elevated serum creatinine (1.59 mg/dL, normal range: 0.7‐1.20). CT scan showed bilateral upper ureteric stones associated with hydronephrosis. He underwent an emergency bilateral JJ stent insertion after which the creatinine returned to normal.

**Figure 1:**
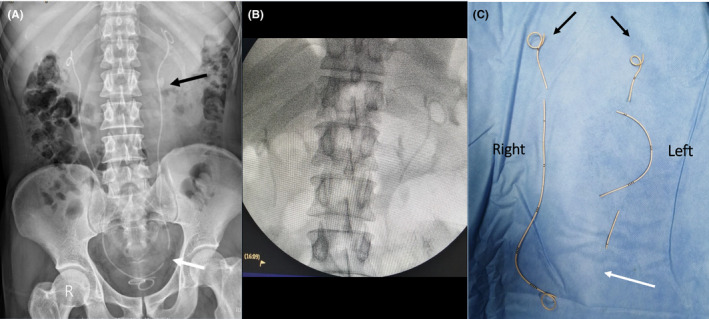
Imaging of the first case. A, Plain x‐ray KUB showing the site of spontaneous fragmentation of the left JJ stent (white arrow). B, Intra‐operative fluoroscopic image showing further fragmentation of the remnant of the left stent. C, Assembly of the retrieved pieces of the fragmented stents following removal. Black arrows indicate the upper end of the stents whereas the white arrow indicates the site of the initial fragmentation of the left stent

When seen at our facility, he admitted having long periods of poor hydration and a very dark urine associated with working outdoors in a very hot environment especially in the past few weeks. Imaging showed a spontaneously fragmented left stent at the level of the lower ureter as shown in Figure 1A. Urinalysis showed pyuria, and there was no bacterial growth. On the following day, he spontaneously passed the lower end of the fragmented stent during voiding. Subsequently, he underwent bilateral ureteroscopy during which the left stent remnant was further spontaneously fragmented into three pieces during removal with a forceps (Figure 1B and C). The right stent was also fragmented into two pieces (Figure 1B and C). During the same session, he underwent bilateral laser lithotripsy for the ureteric stones but the left stone was unintentionally pushed back to the kidney, and therefore, he had bilateral JJ stent insertion. Four weeks later, the left stone was completely fragmented using flexible ureterorenoscopy.

### 2.2 Case‐2

A 32‐year‐old woman who had a history of recurrent stone formation and previous several urological procedures presented to our facility with pelvic pain mainly on the right side and severe dysuria of a few days duration. Reviewing her records revealed a poor dietary control, high protein intake, and high urinary uric acid for long periods. Her body mass index was 41. Five weeks prior to this presentation, she had presented to another healthcare facility with bilateral, mainly left, flank pain associated with fever of two days duration. Imaging at that time showed bilateral small upper ureteric stones with bilateral hydronephrosis and her serum creatinine was 1.65 mg/dl (normal range: 0.7‐1.20). So, she underwent an emergency bilateral JJ stent insertion after which her creatinine returned to normal.

**Figure 2:**
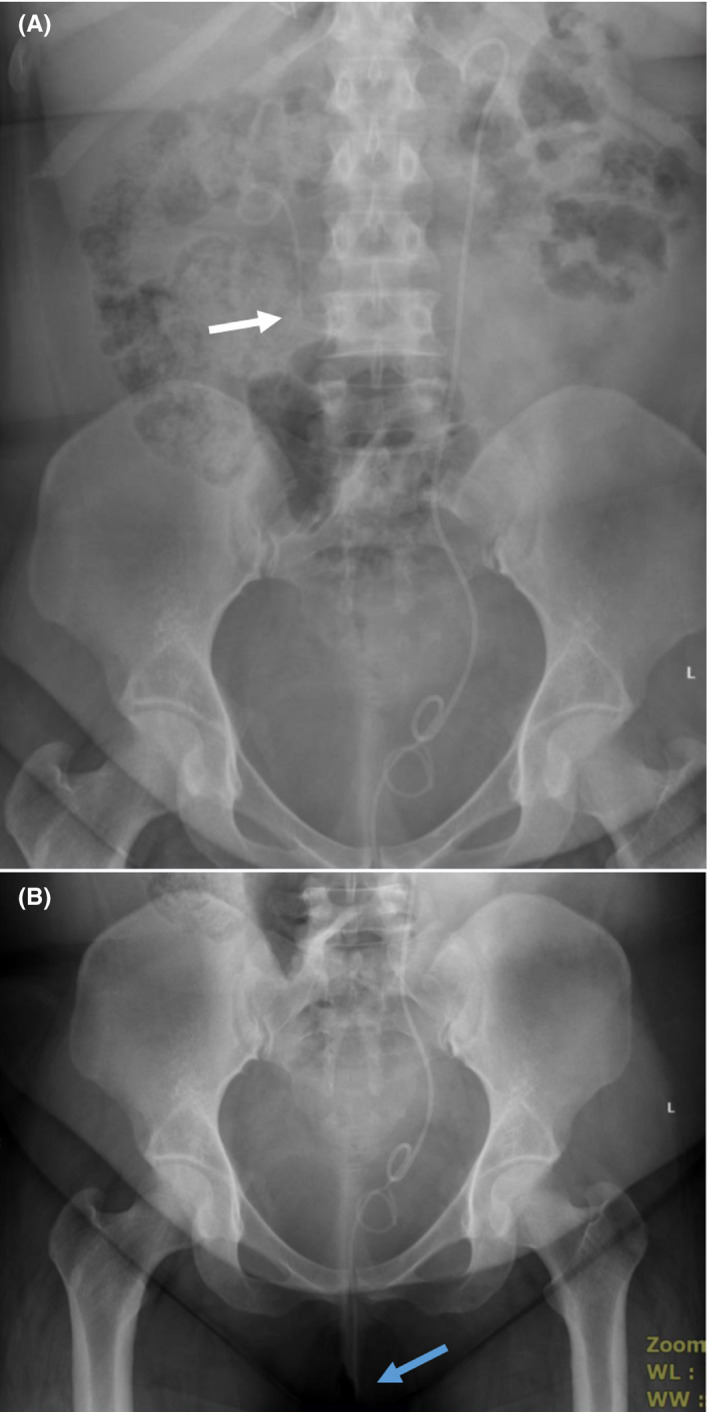
Imaging of the second case. A, Plain x‐ray KUB showing the site of spontaneous fragmentation of the right JJ stent (white arrow). B, Pelvic view showing the straight end of the right stent fragment passing through the urethra (white arrow)

At our facility, a plain x‐ray of the abdomen showed a fragmented left stent at the level of L4 (Figure 2A). The tip of the straight end of the lower fragment was protruding out of the external urethral meatus (Figure 2B). So, this piece was removed in the emergency room. Urinalysis showed pyuria, and there was no bacterial growth. Subsequently, she underwent bilateral ureteroscopy. On the left side, the stent was removed and the ureteric stone was fragmented. Right rigid ureteroscopy showed no ureteric stones, and removal of the upper fragment of the stent using forceps has failed, and this piece was unintentionally pushed back to the renal pelvis. This was removed using flexible ureterorenoscopy and a basket.

## 3 DISCUSSION AND CONCLUSION

In this study, we report for the first time on SSF, which occurred as early as 3 weeks following stent insertion. SSF is a very rare complication.^4^, ^5^, ^6^, ^9^ Regardless of the exact cause of stent fragmentation, both in vivo and in vitro reports have indicated that stents are more likely to fragment across the side drainage holes.^2^, ^13^

SSF has been associated with several potential etiological factors such as the material from which the stent is made. The majority of JJ stents used in common practice are made either completely (like the stents presented in this report) or partly from polyurethane but other materials include silicone, C‐Flex, Percuflex, and metals have also been used.^11^, ^12^ Polyurethane stents are more likely to fragment compared to those made from silicone and other biomaterials.^12^

Aging of the stent has also been recognized as an important cause for stent fragmentation.^10^, ^14^ In this regard, reviewing the literature indicated that SSF had occurred in stents which have been neglected or forgotten for long periods ranging between one year to as long as 15 years which led to stent encrustations and crystal formation.^4^, ^5^, ^6^, ^9^ There was only one report of SSF which occurred after two months of insertion.^8^ In the current study, this has occurred as early as 3 weeks post‐insertion with no evidence of encrustation. The exact reason for this early SSF is difficult to ascertain from the current report but it is unlikely to be due to stent encrustation and calcification like the previous reports.^4^, ^5^, ^6^, ^9^ One possible cause is the presence of a very hostile urine environment in these two patients. The first patient was a construction laborer who works outside in a very hot environment and had long periods of dehydration. The second one was a recurrent stone former with very poor dietary control. In this regard, a hostile urine environment caused by the presence of infection, certain urine constituents, and urine acidity ^7^, ^10^ has been suggested to cause SSF. Indeed, Zisman et al have shown that exposure of the stent to a harsh urine environment had led to increased stent fragility and stiffness.^10^ Regardless of the exact reason, this report indicates that a more strict dietary control including adequate hydration which improves the urine environment and decreases the risk of infection and a close follow‐up including frequent urinalysis are required in specific patients with potential hostile urine characteristics.

Obviously, SSF can be a serious problem, which adds to the patient's morbidity and requires further procedures to remove the fragments. In complex cases especially those with neglected calcified and encrusted stents, more invasive procedures such as percutaneous approach or even open surgery have been used to remove the fragments.^3^, ^10^ Due to the absence of stent encrustations or calcifications in our cases, it was possible to retrieve the stents in a single session using rigid or flexible ureterorenoscopy or a combination of both.

In conclusion, herein we report on two cases of SSF which occurred as early as 3 and 5 weeks post‐insertion. This early fragmentation could be due to a hostile urine environment. To avoid such complications, we suggest a strict dietary modification, adequate hydration, and a close follow‐up.

## CONFLICT OF INTEREST

None declared.

## AUTHOR CONTRIBUTIONS

FH: performed the procedures and, for the two cases, did literature review, consented patents, collected data, and wrote the manuscript.

## ETHICS APPROVAL AND CONSENT TO PARTICIPATE

Both patients had consented for publications, and the report met the local ethics committee regulations (Mediclinic Ethical Committee).

## CONSENT FOR PUBLICATION

Both patients had consented for publications in a written format.

## Data Availability

All imaging is available for review and has been presented.
